# Association between Testicular Cancer and Epididymoorchitis: A Population-Based Case-Control Study

**DOI:** 10.1038/srep23079

**Published:** 2016-03-15

**Authors:** Li-Ting Kao, Herng-Ching Lin, Shiu-Dong Chung, Chao-Yuan Huang

**Affiliations:** 1 Graduate Institute of Life Science, National Defense Medical Center, Taipei, Taiwan; 2 Sleep Research Center, Taipei Medical University Hospital, Taipei, Taiwan; 3 Division of Urology, Department of Surgery, Far Eastern Memorial Hospital, Banciao, Taipei, Taiwan; 4 School of Public Health, Taipei Medical University, Taipei, Taiwan; 5 Department of Urology, National Taiwan University Hospital, College of Medicine National Taiwan University, Taipei, Taiwan

## Abstract

Even though epididymoorchitis and testicular cancer (TC) may have similar pathophysiological pathways, no prior study has attempted to determine the association between these two diseases. This case-control study investigated the relationship between TC and prior epididymoorchitis by using a large population-based database. We used the Taiwan Longitudinal Health Insurance Database 2005 to select 372 patients who had received a diagnosis of TC and 3,720 age-matched controls without TC. We found that of the total sample of 4,092 patients, 53 (1.3%) had received a diagnosis of epididymoorchitis prior to the index date. Patients with TC had a higher prevalence of prior epididymoorchitis than that of patients without TC (11.0% vs. 0.3%, *p* < 0.001). Conditional logistic regression showed that prior epididymoorchitis was significantly associated with TC (crude OR = 38.24, 95% CI = 19.91–73.46). The association remained statistically significant even after adjustment for the other variables (OR = 47.17, 95% CI = 23.83–93.40). This study showed that patients with TC have higher odds of prior epididymoorchitis than do those without TC even after adjustment for potential confounders.

Testicular cancer (TC) is the commonest malignancy among young males, and its incidence has been increasing in many countries^1^. Although the pathophysiology of TC is still controversial, various studies have reported that TC might have multifactorial etiologies, such as cryptorchidism, inguinal hernia, birth order, and sibship^2^,^3^,^4^. In addition, there is increasing biological and epidemiologic evidence that some infections and infectious agents might increase the risk of TC^5^,^6^,^7^,^8^,^9^,^10^. Many authors have suggested that inflammatory mechanisms may promote cancer development^11^,^12^. Thus, infections and inflammation may play important roles in the occurrence and progression of TC. However, to date, very few studies have explored the relationship between prevalent genitourinary infections and TC.

Epididymoorchitis is a common inflammatory disease that affects the epididymis and the testis^13^,^14^. It usually occurs in men between 18 and 35 years of age. Every year, approximately 600,000 patients in the United States experience the symptoms of epididymoorchitis^14^. Previous studies have shown that epididymoorchitis can be caused by a wide range of infections, including sexually transmitted infections, urinary infections, and viral infections^13^,^14^,^15^,^16^. These infections and the associated inflammation have been considered to be involved in the etiology of TC.

Even though epididymoorchitis and TC may have similar infectious and inflammatory pathophysiological pathways, no prior study has attempted to determine the association between these two diseases. Therefore, we designed this case-control study to investigate the relationship between epididymoorchitis and TC by using a large population-based database in Taiwan.

## Materials and Methods

### Database

We retrieved the study sample from the Longitudinal Health Insurance Database 2005 (LHID2005), which was created by the Taiwan National Health Research Institutes. Taiwan initiated the single-payer National Health Insurance (NHI) program in 1995 to provide accessible and affordable healthcare for all residents. The LHID2005 includes all medical claims data for 1,000,000 beneficiaries, randomly sampled from among all enrollees (*n* = 25.68 million) in the NHI program. The LHID2005 provides a unique opportunity for researchers to trace the medical services availed of by these 1,000,000 beneficiaries since the beginning of the NHI program in 1995.

### Selection of Cases and Controls

From the LHID2005, we identified 372 patients who had received a first-time diagnosis of TC (ICD-9-CM codes 186, 186.0, and 186.9) in ambulatory care visits (outpatient department of hospitals or clinics) or hospitalization between January 2001 and December 2013. We ensured that in all cases the diagnosis had been verified by a certified urologist on at least one occasion. The date of the first diagnosis of TC was denoted the index date for that patient.

The controls were also selected from the LHID2005. We first excluded all subjects who had a history of TC. Subsequently, using the SAS PROC SURVEYSELECT program (SAS System for Windows, Version 8.2), we selected 3,720 controls, in a ratio of 10:1. The ten controls per case were matched according to age group (<18, 18–29, 30–39, 40–49, 50–59, and >59 years) and the index year; specifically, for each TC case, the controls were selected from among those who had utilized medical services in the index year of that particular case. The date of the first use of ambulatory care visits occurring in the index year was designated as the index date for the controls.

### Exposure Assessment

From the medical claims data, we identified all patients who had received a diagnosis of epididymoorchitis (ICD-9-CM codes 604, 604.0, and 604.9) during ambulatory care visits at any time in the 3 years preceding the index date. We confirmed that the diagnoses were made by certified urologists.

### Statistical Analysis

We used the SAS statistical package (SAS System for Windows, Version 8.2) for data analysis. Independent t test and Chi-square test were conducted to compare differences between the cases and controls in terms of the patients’ age, monthly income, geographic location, and urbanization level (five levels, with 1 being the most urbanized and 5 being the least). In addition, we explored the differences in medical comorbidities between the cases and controls by using the Pearson χ^2^ test. The comorbidities were inguinal hernia, cryptorchidism, testicular microlithiasis, and mumps orchitis, which have been identified by previous studies as potential risk factors for TC. These comorbidities were counted only if they were coded prior to the index date. We used conditional logistic regression (conditioned on the factors of age group and index year) to calculate the odds ratio (OR) and 95% confidence interval (CI) for having previously received an epididymoorchitis diagnosis between the cases and controls. A two-sided *p* value ≤0.05 was considered significant.

## Results

Table 1 shows the distribution of the sociodemographic characteristics and comorbidities among the cases and controls. There were no significant differences in the mean age (*p* = 0.865) and monthly insured amount (*p* = 0.970) between the cases and controls. However, there were significant differences in urbanization and geographic region (*p* < 0.001). In addition, testicular microlithiasis was significantly more common in the cases than in the controls (*p* < 0.001).

Table 2 presents the relationship between TC and epididymoorchitis. Of the total sample of 4,092 patients, 53 (1.3%) had received a diagnosis of epididymoorchitis prior to the index date—41/372 (11.0%) cases and 12/3,720 (0.3%) controls. The chi-square test showed a statistically significant relationship between TC and prior epididymoorchitis (*p* < 0.001).

Table 3 shows the crude and adjusted ORs for TC. Conditional logistic regression revealed that prior epididymoorchitis was significantly associated with TC (crude OR = 38.24, 95% CI = 19.91–73.46); this association remained statistically significant even after adjustment for testicular microlithiasis, geographic region and urbanization level (OR = 47.17, 95% CI = 23.83–93.40). Furthermore, after adjustment for the other variables, testicular microlithiasis was significantly associated with TC (adjusted OR = 5.88, 95% CI = 2.77–12.48).

## Discussion

This population-based case-control study showed that prior epididymoorchitis was significantly associated with TC even after adjustment for some potential confounders. Many previous studies have investigated the association between TC and factors such as cryptorchidism, inguinal hernia, birth order, and infertility^2^,^3^,^4^,^17^,^18^,^19^. Epidemiologic studies and meta-analysis have also attempted to demonstrate the relationship between TC and mumps orchitis^2^,^7^,^8^,^20^,^21^. However, in our review of the literature, we could not find any study that has examined the association between TC and prior epididymoorchitis; this is surprising because these two diseases might have similar pathophysiological mechanisms, and epididymoorchitis is more common than isolated orchitis^14^.

The mechanisms underlying the relationship between epididymoorchitis and TC are still uncertain. The greater odds of prior epididymoorchitis in patients with TC in comparison with those without TC might be explained by the infection and subsequent inflammatory mechanisms. Some studies have documented the potential connection between TC and infections^5^,^9^,^10^,^22^,^23^,^24^. A study in the 1980s was the first to suggest an infectious etiology for TC because of its epidemiologic similarities with Hodgkin disease^22^. Since then, increasing biological and epidemiologic evidence has supported the possibility that some infectious agents might increase the risk of the development of TC. For instance, one study reported a significantly increased risk of TC among patients infected with human immunodeficiency virus^23^. Other studies have suggested that the Epstein-Barr virus and the cytomegalovirus may be involved in the etiology of TC^5^,^9^,^10^,^24^. Studies in the United Kingdom have indicated an elevated risk of TC in patients with a history of sexually transmitted disease or *Neisseria gonorrhoeae* infection^6^,^7^. Epididymoorchitis is mostly an infectious disease that is caused by a broad range of infections, including sexually transmitted infections (such as infection with *N. gonorrhoeae* and *Chlamydia trachomatis*), urinary tract infections, and other bacterial and viral infections^14^,^15^,^25^,^26^. Therefore, these pathogens and the infectious process have been considered to be involved in the etiology of both epididymoorchitis and TC.

The inflammatory process associated with infections may also be a major factor in TC development. According to the literature, epididymoorchitis is a frequent cause of scrotal pain, which is an indication of inflammation of the epididymis and testis^14^,^25^. Epididymoorchitis elicits inflammatory responses, such as increases in the number of leukocytes and levels of proinflammatory cytokines^27^. Many recent studies have shown that inflammation may play a key role in cancer growth and progression^12^,^28^,^29^. The chronic inflammation associated with infections could promote tumor development^29^. In addition, some tumors have been reported to arise from areas of infections and inflammation^11^. Therefore, the abnormal inflammatory responses in patients with epididymoorchitis might lead to the progression and exacerbation of TC.

The particular strength of this case-control study was the use of the LHID2005—a large population-based dataset with wide health benefit coverage in Taiwan. This enabled us to obtain a large sample and thus increase the statistical power of the study. Use of this database helped us avoid any selection bias and, because the LHID2005 includes most of the relevant medical data for selected patients since they entered the healthcare system in Taiwan, we could eliminate the possibility of a recall bias.

There are, however, several limitations that must be considered. First, the LHID2005 does not provide information on bacterial cultures and the severity of TC (e.g., TNM classification), which are considered to affect the potential association between epididymoorchitis and TC^10^,^30^. Second, this administrative dataset does not provide information on genetic factors, maternal risk factors, and lifestyle factors, including a family history of cancer, maternal bleeding, and cigarette smoking and therefore we could not adjust for these confounding factors in this study^4^,^10^,^30^. Third, although the LHID2005 is a population-based dataset, it might not include all patients who have had epididymoorchitis in Taiwan; several patients with mild symptoms might not have sought medical treatment. Finally, most of the selected patients in this study were of Chinese ethnicity and therefore the generalization of the findings to other ethnic groups is not possible.

In conclusion, this population-based case-control study shows that patients with TC had higher odds of prior epididymoorchitis than did those without TC even after adjustment for potential confounders. We recommend that physicians be alert to this relationship in patients with a history of epididymoorchitis and recommend regular urological examinations for such patients. Additional biological studies are required to define the actual mechanisms underlying the association between epididymoorchitis and TC.

## Additional Information

**How to cite this article**: Kao, L.-T. *et al*. Association between Testicular Cancer and Epididymoorchitis: A Population-Based Case-Control Study. *Sci. Rep.* **6**, 23079; doi: 10.1038/srep23079 (2016).

## Figures and Tables

**Table 1:** Sociodemographic characteristics and medical comorbidities of testicular cancer patients and controls (*n* = 4,092).

	Patients with Testicular Cancer *n* = 372		Controls *n* = 3,720		
Variables	No.	(%)	No.	(%)	*p* value
Age, mean (standard deviation)	34.4 (18.9)	34.2 (18.9)	0.865		
Monthly insured income					0.970
≤NT$15,840	194	(52.2)	1,932	(51.9)	
NT$15,841-25,000	82	(22.0)	840	(22.6)	
≥NT$25,001	96	(25.8)	948	(25.5)	
Urbanization level					<0.001
1	90	(24.2)	1,158	(31.1)	
2	118	(31.7)	1,058	(28.4)	
3	39	(10.5)	638	(17.2)	
4	45	(12.1)	468	(12.6)	
5	80	(21.5)	398	(10.7)	
Geographic region					<0.001
Northern	14,321	(37.9)	1,844	(49.6)	
Central	182	(48.9)	906	(24.4)	
Southern	45	(12.1)	(920	(24.7)	
Eastern	4	(1.1)	50	(1.3)	
Inguinal hernia	13	(3.5)	106	(2.9)	0.480
Cryptorchidism	2	(0.5)	10	(0.3)	0.361
Testicular microlithiasis	15	(4.0)	22	(0.6)	<0.001
Mumps orchitis	0	(0)	2	(0.1)	--

**Table 2:** Relationship between testicular cancer and prior epididymoorchitis (*n* = 4092).

Presence of Prior Epididymoorchitis	Patients with Testicular Cancer *n* = 372		Controls *n* = 3720		
	No.	%	No.	%	*p* value
Yes	41	(11.0)	12	(0.3)	<0.001
No	331	(89.0)	3,708	(99.7)	

**Table 3:** Crude and adjusted odds ratio for testicular cancer (*n* = 4092).

Variables	Testicular Cancer Occurrences			
	Crude OR (95% CI)	*p* value	Adjusted OR (95% CI)	*p* value
Prior epididymoorchitis	38.24 (19.91–73.46)	<0.001	47.17 (23.83–93.40)	<0.001
Urbanization level				
1	1.00		1.00	
2	1.44 (1.08–1.91)	0.014	1.29 (0.94–1.77)	0.110
3	0.79 (0.53–1.16)	0.225	0.56 (0.37–0.87)	0.009
4	1.24 (0.85–1.80)	0.264	0.72 (0.47–1.12)	0.145
5	2.59 (1.87–3.57)	<0.001	1.80 (1.24–2.61)	0.002
Geographic region				
Northern	1.00		1.00	
Central	2.63 (2.08–3.32)	<0.001	2.98 (2.26–3.95)	<0.001
Southern	0.64 (0.45–0.90)	0.0111	0.59 (0.40–0.86)	0.006
Eastern	1.05 (0.37–2.94)	0.9316	0.86 (0.27–2.79)	0.801
Testicular microlithiasis	7.06 (3.63–13.74)	<0.001	5.88 (2.77–12.48)	<0.001

Note: The adjusted odds ratios were derived from a conditional logistic regression model and adjusted for potential confounders.
